# Effects of Acupuncture on Cardiac Remodeling in Patients with Persistent Atrial Fibrillation: Results of a Randomized, Placebo-Controlled, Patient- and Assessor-Blinded Pilot Trial and Its Implications for Future Research

**DOI:** 10.3390/medicina58010041

**Published:** 2021-12-27

**Authors:** Jung Myung Lee, Seung Min Kathy Lee, Jungtae Leem, Jin-Bae Kim, Jimin Park, Jun Hyeong Park, Suji Lee, Hyung Oh Kim, Hyemoon Chung, Jong Shin Woo, Woo-Shik Kim, Sanghoon Lee, Weon Kim

**Affiliations:** 1 Division of Cardiology, Department of Internal Medicine, Kyung Hee University Hospital, Kyung Hee University, 23 Kyungheedae-ro, Dongdaemun-gu, Seoul 02447, Korea; cardioljm@gmail.com (J.M.L.); jinbbai@khu.ac.kr (J.-B.K.); hypnotica1999@hanmail.net (H.O.K.); 111212@hanmail.net (H.C.); snowball77@hanmail.net (J.S.W.); kkabee@dreamwiz.com (W.-S.K.); 2 Department of Acupuncture and Moxibustion, Kyung Hee University Korean Medicine Hospital, 23 Kyungheedae-ro, Dongdaemun-gu, Seoul 02447, Korea; lollymin@nate.com (S.M.K.L.); swarmy77@hanmail.net (J.P.); good-iii@hanmail.net (J.H.P.); sjstarry41@naver.com (S.L.); 3 Jaseng Medical Academy, Jaseng Hospital of Korean Medicine, 538 Gangnam-daero, Gangnam-gu, Seoul 06110, Korea; 4 Research Center of Traditional Korean Medicine, Wonkwang University, 460, Iksan-daero, Iksan-si 54538, Korea; julcho@naver.com; 5 Department of Medical Education, College of Korean Medicine, Kyung Hee University, 23 Kyungheedae-ro, Dongdaemun-gu, Seoul 02447, Korea

**Keywords:** acupuncture, medicine, East Asian traditional, atrial fibrillation, randomized controlled trial, flecainide, membrane transport modulator

## Abstract

*Background and Objectives:* In this study, we attempted to determine the effects of acupuncture on cardiac remodeling and atrial fibrillation (AF) recurrence rates in patients with AF after electrical cardioversion (EC). *Materials and Methods:* We randomly assigned 44 patients with persistent AF to an acupuncture group or a sham acupuncture group. An electroacupuncture treatment session was administered once weekly for 12 weeks at four acupuncture points (left PC5, PC6, ST36, and ST37). *Results:* Among the 44 recruited participants, 16 (treatment group) and 15 (control group) completed the trial. The three-month AF recurrence rate (primary outcome) was not significantly different between the two groups. Following the completion of treatment, patients who had been treated with acupuncture had a significant reduction in left atrial volume index (42.2 ± 13.9 to 36.1 ± 9.7 mL/m^2^; *p* = 0.028), whereas no change in atrial size was observed in the sham acupuncture group. No serious adverse events were observed. The AF recurrence rate and cardiac function did not differ significantly between the two groups. At three months, the acupuncture treatment group showed more favorable atrial structural remodeling compared to the sham acupuncture group. *Conclusion:* In future research on acupuncture in AF management, it is recommended that the inclusion criteria be amended to include only symptomatic AF, that an appropriate control group is designed, and that the acupuncture treatment frequency is increased to several times per week.

## 1. Introduction

Atrial fibrillation (AF) is the most prevalent cardiac arrhythmia and is the most frequent cause of cardiac embolic stroke [1,2]. Despite its commonality, management of AF is difficult because of the limited efficacy and safety of current antiarrhythmic drugs, as well as the high recurrence rate of AF after radiofrequency catheter ablation. A recent randomized trial showed that the recurrence rate of AF after single radiofrequency catheter ablation was high, particularly in patients with persistent AF [3]. Even after AF ablation, some patients suffer potentially fatal complications, such as cardiac tamponade and atrioesophageal fistula [4]. Current guidelines recommend electrical cardioversion (EC) for patients with AF unresponsive to antiarrhythmic drugs [1], but the preventive effect of antiarrhythmic drugs on AF recurrence after successful EC is low. Therefore, it is necessary to develop safer and more efficient treatment methods.

Acupuncture is a nonpharmacological therapeutic intervention commonly used in East Asia. Some recent experimental studies have suggested that acupuncture may be beneficial in cases of cardiac arrhythmias, including atrial fibrillation [5,6,7]. It has been reported that patients with persistent AF treated with once-weekly acupuncture over 10 weeks after EC had significantly less AF recurrence than the control group. Additionally, the acupuncture group of patients with paroxysmal AF had a significant reduction in the number of AF episodes [5,8]. More recently, Yin et al. reported that seven consecutive days of manual acupuncture therapy for hospitalized patients reduced the early recurrence rate [8]. Despite these promising results, no previous studies have attempted to assess cardiac function.

The effects of acupuncture on autonomic function may illuminate the mechanism through which it exerts protective effects in patients with cardiac arrhythmias. Stravrakis et al. recently suggested that stimulation of the autonomic nervous system via the tragus can significantly affect cardiac electrophysiology [9]. Interestingly, the area of this tragus stimulation was near the traditional auricular acupuncture points. Another recent small prospective randomized study reported that acupuncture at the PC6, HT7, and BL15 points may significantly reduce AF recurrence in patients with persistent AF after cardioversion [10].

The effect of EA on AF recurrence and cardiac function has yet to be studied in an outpatient setting. Therefore, we conducted a double-blinded randomized controlled trial to determine the effects of EA on AF recurrence, cardiac remodeling, and cardiac function in an outpatient department setting.

## 2. Materials and Methods

### 2.1. Trial Design

This study was a single-center, prospective, participant- and assessor-blinded, randomized, sham-controlled, and two-parallel-arm clinical trial. A detailed description of these methods has been previously published [11]. Our trial was conducted at a tertiary hospital in Seoul, Republic of Korea, which has both a conventional cardiology department and a traditional Korean medicine department.

Forty-four patients who were resistant to two weeks of flecainide therapy were randomized into active or sham acupuncture groups (22 patients in each group) at a ratio of 1:1 by central randomization. Acupuncture was provided for two weeks before the EC. Simultaneous anticoagulation therapy with warfarin was performed before EC and during the follow-up period, in accordance with the current guidelines. Transesophageal echocardiography (TEE) was performed just before EC to rule out atrial thrombus. EC was conducted using direct-current electric shocks. Sodium pentothal (1.5 mg/kg) was used for patient sedation before the procedure. Administration of 75 mg of flecainide twice daily was maintained during the follow-up period in both groups.

**Figure 1:**
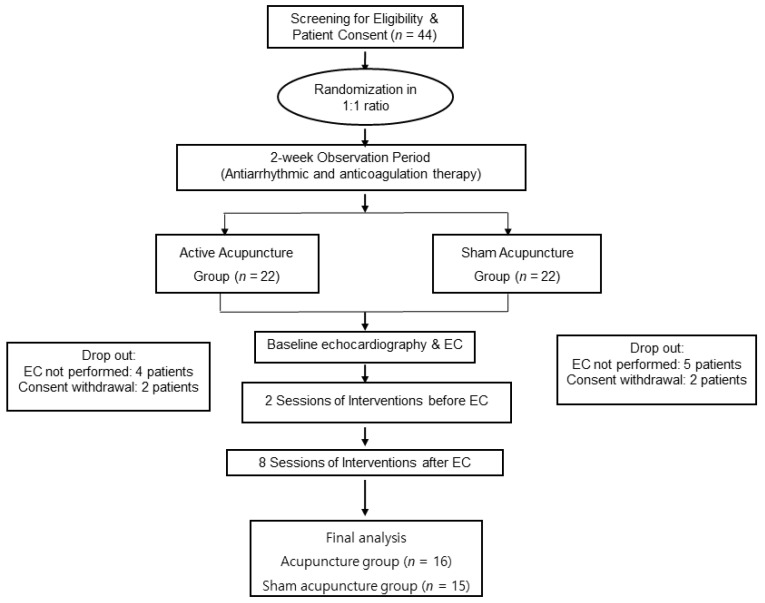
Trial flow diagram of acupuncture in persistent AF. Patients received active acupuncture or sham acupuncture treatment according to the assigned sequence and AF recurrence was assessed. AF recurrence was evaluated using 48-h Holter monitoring, electrocardiography, and follow-up echocardiography. AF: Atrial fibrillation, EC: Electrical cardioversion, ECG: Electrocardiography, LA: Left atrium, LAA: Left atrial appendage.

After EC, the participants continued to receive active or sham acupuncture treatment once per week for the next 10 weeks and were permitted to receive heart rate-control drugs. A flowchart of the study is presented in Figure 1.

### 2.2. Randomization and Blinding

We used a computer-generated random number table with a permuted block design at a 1:1 ratio. After obtaining consent, the independent clinical research coordinator allocated the patients according to a random number table. The coordinator contacted the acupuncture practitioner by telephone to inform them of the group allocation. As we used a sham acupuncture design, the acupuncture treatment procedure was similar, except for the treatment location. Therefore, the patients were blinded to the study. The assessors, sonographers, and statisticians were blinded. The acupuncture practitioner could not be blinded to the characteristics of the sham acupuncture design, as they needed to know the exact treatment location.

### 2.3. Participants

Acupuncture treatment during EC was conducted between February 2014 and June 2017 at the Cardiology Department of Kyung Hee University Hospital. Consecutive patients with persistent AF refractory to class Ic antiarrhythmic drug therapy (flecainide 150 mg/day) were enrolled in this trial. The exclusion criteria for acupuncture treatment were as follows: (1) age ˂ 20 years or ˃75 years; (2) severe valvular heart disease; (3) history of open heart surgery; (4) treatment for myocardial infarction (MI) in the past six weeks; (5) administration of, or need for antiviral agents; (6) second-degree atrioventricular block or more than two fascicular blocks; (7) severe pulmonary, liver, or renal disease; (8) acupuncture treatment for a cardiovascular condition in the last three months; and (9) contraindication to flecainide. The CHA_2_DS_2_VASC score (1 point for congestive heart failure, 1 point for hypertension, 2 points for age ≥75 years, 1 point for diabetes mellitus, 2 points for stroke or transient ischemic attack, 1 point for vascular disease, 1 point for age ≥65 years, 1 point for female sex) was calculated for each patient. As there were not enough previous studies to refer to, the inclusion and exclusion criteria were selected based on the agreement of the research team. We also excluded patients with secondary AF, active infection, active thyroid disease, and severe pulmonary/renal/liver disease. Additional detailed exclusion criteria are described in our previously published protocol [11]. Written informed consent was obtained from all patients before acupuncture treatment.

### 2.4. EC Protocol

After sedation with pentobarbital, synchronized 150 J biphasic electric shock EC was performed using direct-current electric shocks. One electrode pad was placed in the second intercostal space to the right of the sternum, and the other was placed in the fifth intercostal space near the mid-axillary line. EC was initiated as a 100 J biphasic waveform shock and increased to 200 J until restoration of sinus rhythm. If conversion to sinus rhythm occurred, the patient was administered eight additional once-per-week acupuncture interventions. If conversion to sinus rhythm did not occur, the patient was excluded from the study. Dropped patients received other standard treatments and were not included in this study because acupuncture was not considered.

### 2.5. Outcomes

#### 2.5.1. Primary Outcome

The primary outcome of our study was the recurrence rate of AF after EC. Three months after EC, Holter monitoring was conducted for 48 h to determine AF recurrence. AF recurrence was defined as AF episodes exceeding 30 s via Holter monitoring or documentation of AF on a 12-lead ECG.

#### 2.5.2. Secondary Outcome

Echocardiography was performed to assess cardiac function and remodeling. Comprehensive echo/Doppler evaluations were performed before and three months after cardioversion. Left ventricular (LV) wall thickness was measured in the end-diastolic phase. All measurements were performed in accordance with the current guidelines [12]. The modified Simpson’s rule was used to calculate LV volumes and LV ejection fraction (LVEF) from apical 2- and 4-chamber views. The volumetric method was used to calculate the left atrial (LA) volume from apical 4- and 2-chamber views at ventricular end-systole, and the LA volumes were then indexed to the body surface area. Peak early (E) and late (A) diastolic mitral inflow velocities were measured in the apical four-chamber view. Tissue Doppler interrogation was performed at the septal mitral annulus in the apical four-chamber view. Peak systolic mitral annulus velocity and early diastolic mitral annulus peak velocity (E’) were then measured and the ratio E/E’ was calculated. Pulsed Doppler and pulsed tissue Doppler parameters were measured as the average of three cardiac cycles, and the R-R intervals were relatively regular except during long-lasting AF. TEE was performed just before EC to rule out atrial thrombus. As reported in previous studies, the anticipated adverse events were bleeding, hematoma, pain, nerve injury, weakness, fatigue, sleepiness, dizziness, headache, nausea, and any other symptoms that patients wished to share. We evaluated these events at every patient visit during the treatment and follow-up periods.

### 2.6. Intervention

#### 2.6.1. Real Acupuncture Treatment

Two forms of acupuncture, electroacupuncture (EA) and intradermal acupuncture (IDA), were available for the real acupuncture treatment group.

**Figure 2:**
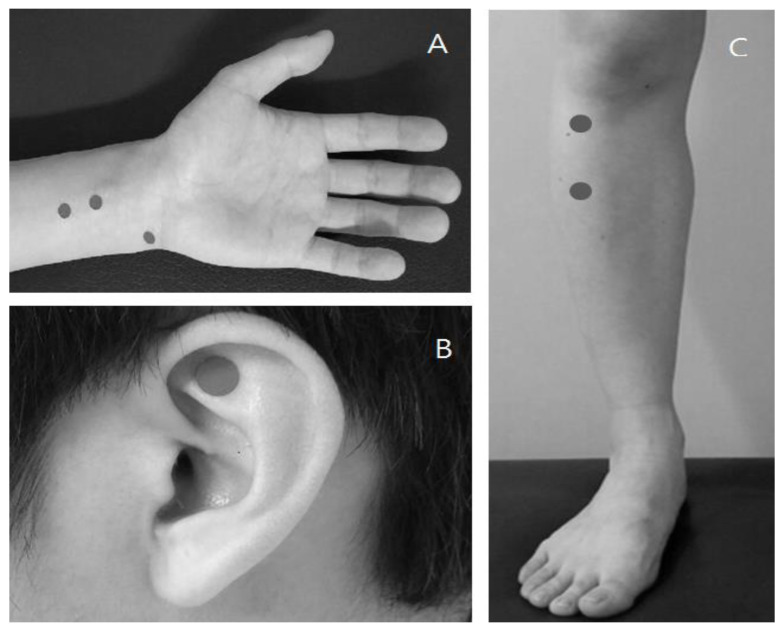
Location of acupuncture points. (**A**) PC5, PC6, HT7; (**B**) ST36, ST37; and (**C**) TF4.

EA treatment entailed unilateral insertion of needles at four acupuncture points (left side PC5, left side PC6, right side ST36, and right side ST37; Figure 2A,B) [13]. Disposable sterile needles (0.2 × 30 mm, Dongbang Acupuncture Inc., Boryung, Korea) were inserted to a depth of 2 ± 0.5 cm at a 90-degree angle and EA clips were connected to PC5-PC6 and ST36-ST37. A continuous electric current of 2 Hz was applied using a low-frequency electrical stimulator (ES-160, ITO, Tokyo, Japan). It is important to note that before the application of electric current, the acupuncture needles were rotated several times to provoke de-qi. Deqi is a characteristic sensation of numbness, heaviness, soreness, or distension, and its induction is known to increase the effects of acupuncture. EA intensity was regulated so that the patient could feel a slight twitching sensation, but not to the point of causing discomfort. The treatment lasted for 20 min.

IDA treatment entailed the bilateral insertion of disposable sterile sticker-type needles (0.18 × 1.3 × 1.5 mm, Dongbang Acupuncture Inc., Boryung, Korea) at two acupuncture points (HT7 and TF4—the Shen Men points), making a total of four acupuncture points [13]. Each sticker-type needle was inserted at a depth of 1 mm at a 90-degree angle. The sticker-type needles were maintained for as long as possible, without the patient feeling of discomfort or pain. The patients were taught to repetitively stimulate the needles by pressing them on the stickers.

The acupuncture treatments were administered by practitioners who had completed six years of training as doctors of Korean medicine at the College of Korean Medicine. All of the practitioners were licensed by the Korean Ministry of Health and Welfare, had >3 years of clinical experience, and were trained to follow the study protocol (Tables S1 and S2).

#### 2.6.2. Sham Acupuncture Treatment

The sham treatment entailed superficial stimulation at non-acupuncture points. As with the active treatment, two types of sham acupuncture were performed, namely sham EA and sham IDA. In the sham EA group, four non-acupuncture points were chosen. These points were located 1.5–2 cm lateral to the real acupuncture points and the insertions were made to a depth of 0.5 cm at a 90-degree angle. To make the patients feel as though they were receiving active EA treatment, EA clips were connected to the acupuncture needles and the same clicking sounds were induced from the machine. De-qi sensation was not induced, and no electric current was applied. In the sham IDA treatment, four non-acupuncture points were selected, and needles were inserted just like in the active IDA treatment. These results are listed in Table S2. The patients were also taught to repetitively stimulate the needles. All of the study parameters, such as needle size, retention time, frequency, number of treatments, and the attending practitioner, were identical in the active and sham acupuncture groups (Tables S1 and S2).

### 2.7. Sample Size

In our previously published protocol, the calculated sample size for each group was 40 [11]. According to a previous study, the AF recurrence rate was 35.3% in the treatment group and 69.2% in the control group. We calculated the sample size based on a 5% significance and 80% power with a two-tailed analysis. We anticipated a dropout rate of 20%.

### 2.8. Statistical Analysis

Normally distributed continuous variables were expressed as mean ± standard deviation and compared using the Student’s *t*-test. Data that were not normally distributed according to the normality test were expressed as median (interquartile range), and the nonparametric method (Wilcoxon signed rank test) was used to compare echocardiographic parameters before and after acupuncture treatment. Categorical variables are expressed as frequencies and percentages and were compared using the chi-squared test or Fisher’s exact test, as appropriate. Statistical significance was set at *p* < 0.05. Statistical analysis was performed using R software 3.5.2 version (R Foundation for Statistical Computing, Vienna, Austria).

### 2.9. Ethical Considerations

This study was approved by the Institutional Review Board (IRB) of the Kyung Hee University Medical Center (IRB number: KMMC 1335-04) and was conducted in accordance with the Declaration of Helsinki. The study protocol was carefully explained to the participants in detail, and written informed consent was obtained from all participants. This study was registered with clinicaltrials.gov (NCT02110537). CONSORT 2010 checklist are provided in Table S3.

## 3. Results

### 3.1. Participant Flow and Recruitment

Although we planned to recruit more patients, patient recruitment was difficult, and we were unable to finish recruiting before the funding body’s deadline, as well as our planned research period. Therefore, we abruptly finished recruitment and began the analysis with a total of 80 recruits. Between February 2014 and June 2017, 44 patients (22 patients in each group) were included in this study. Four patients in the active acupuncture group and five patients in the sham acupuncture group were excluded because they did not undergo EC. Two patients from the active acupuncture and sham acupuncture groups withdrew their consent and were excluded from the study. Thus, for final data analysis, 16 and 15 patients in the active acupuncture and sham acupuncture groups, respectively, were included.

### 3.2. Baseline Characteristics

The baseline characteristics of the study population are summarized in Table 1. Patients randomized to the active acupuncture group were older (69.6 ± 7.3 vs. 63.7 ± 6.5 years; *p* = 0.024) and had higher CHA_2_DS_2_VASC scores (2.8 ± 1.5 vs. 1.6 ± 1.0; *p* = 0.02) than patients randomized to the sham acupuncture group. There was no significant difference in the frequency of medication use between the two groups. The proportions of comorbidities, such as hypertension and diabetes mellitus, were not significantly different across the study groups. Furthermore, LA size, LVEF, and mitral inflow velocity over septal mitral annulus tissue velocity (E/E’) values did not differ between the study groups.

### 3.3. Primary Outcome

We assessed the AF recurrence rate at the three-month follow-up. In the real acupuncture group, AF recurred in 12 (80%) of the 16 patients. In the sham acupuncture group, AF recurred in nine (60%) of the 15 patients. The recurrence rates were not significantly different between the two groups (*p* = 0.611; Table 2).

### 3.4. Secondary Outcomes

**Figure 3:**
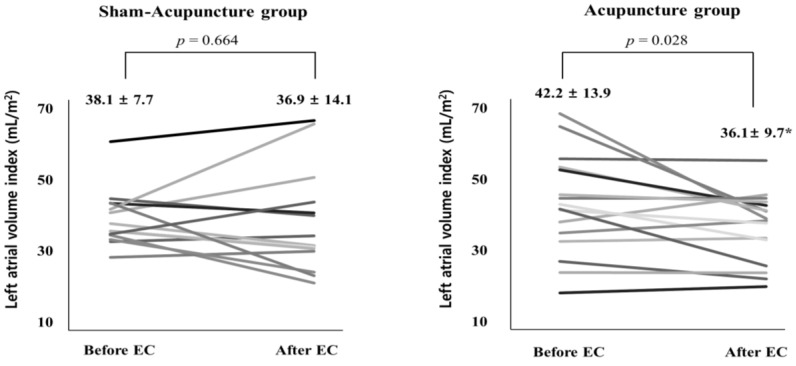
Change in left atrial size in patients of the acupuncture treatment group. * *p* < 0.05 compared with baseline.

Compared with the baseline value, the LA size (LAVI) in the patients treated with acupuncture was significantly reduced (from 42.2 ± 13.9 to 36.1 ± 9.7 mL/m^2^; *p* = 0.028; Table 1 and Table 2). In contrast, there was no significant change in LAVI before and after EC in patients of the sham acupuncture group (from 38.1 ± 7.7 to 36.9 ± 14.1; *p* = 0.664; Table 1 and Table 2). Figure 3 shows the change in LA volume index at the individual patient level and a clear trend of decreasing LA volume in patients treated with acupuncture. In logistic regression analysis, acupuncture treatment did not predict AF recurrence at three months (data not shown).

No adverse and serious adverse events occurred in either of the two groups.

## 4. Discussion

### 4.1. Summary of Findings

In this study, we found no significant short-term preventive effects of acupuncture after EC on AF recurrence. However, acupuncture treatment resulted in favorable cardiac structural remodeling, and patients treated with acupuncture showed a significant reduction in the LA volume index.

### 4.2. Discussion

Several systematic reviews have reported that acupuncture treatment may yield positive results in patients with AF after cardioversion, with a reduced incidence of adverse events [14,15]. The possible mechanism of action was explained by the ability of acupuncture to reduce sympathetic outflow and normalize parasympathetic activity [14,15,16,17]. Among the acupuncture points that were previously explored, PC6 was especially significant in inhibiting sympathetic tone, thereby lowering the heart rate and systolic blood pressure [18]. In our study, HT7 and ST36 were used alongside PC6 because these points are well known to have cardiological effects [6,10]. IDA treatment has not been explored in previous studies. The points on the ear are similar in area to tragus stimulation, and TF4 was used to stimulate the autonomic nervous system.

This study revealed that patients who received active acupuncture treatment had more favorable atrial structural remodeling than those who received sham acupuncture, with no severe adverse events observed in either group. Atrial remodeling is defined as any change in atrial structure or function that promotes atrial arrhythmias, and AF can promote atrial remodeling. In the clinic, these changes are generally difficult to assess, but structural remodeling can be identified relatively easily using echocardiography. Four principal pathophysiological mechanisms contribute to AF: electrical remodeling, structural remodeling, Ca^2+^ handling abnormalities, and autonomic nervous system changes [16].

Atrial enlargement is a principal component of atrial structural remodeling, and is a key determinant of the persistence of AF. Although echocardiography cannot assess atrial fibrosis, the echocardiographic parameters are significantly associated with atrial fibrosis. In the Framingham Heart Study, the increment in LA size was found to be an independent echocardiographic predictor of the future development of AF [17]. Earlier reports clearly showed that increased LA size is an important prognostic factor for death, MI, AF, and stroke [18,19]. Changes in the autonomic nervous system are an important part of the pathophysiology of atrial fibrillation. Sympathetic activation can increase L-type calcium current, ryanodine receptors, and sarcoplasmic reticulum Ca^2+^ via Ca^2+^/calmodulin-dependent protein kinase II and protein kinase A [16]. Consequently, these changes may affect not only the electrical remodeling of the atrium, but also structural remodeling.

After restoration of the sinus rhythm, the atria of patients with AF show some degree of improvement in structure and/or function. This is referred to as atrial “reverse” remodeling. Reverse remodeling, which is measured by LA volume, has been associated with fewer AF recurrences after catheter ablation or surgery for rhythm control in patients with AF. It has been suggested that the blockade of the renin-angiotensin system may be related to atrial reverse remodeling [20].

In an animal study, angiotensin blockade did not produce a difference in action potential parameters and the effective refractory period, but did reduce structural remodeling of the left atrium [21]. Therefore, reducing the load of AF is not the only way to reduce the structural remodeling of the atrium, and additional methods, such as blocking neurohormonal activation, can also reverse remodeling. As acupuncture significantly reduced the sympathetic outflow, it may have also led to inverse remodeling of the atrium, although further studies are needed to determine this.

In terms of participants, our population showed moderate LA structural remodeling according to the LV volume index. If we had selected patients with larger LA, the effect of acupuncture may have been more pronounced because the decrease in LA size would be more easily detectable by TTE. However, if we had selected a population with a large LA, LA remodeling might be irreversible by acupuncture. The exact timing at which atrial remodeling becomes irreversible is not known.

Three months of EA treatment on the left PC5, PC6, ST36, ST37, and acupuncture on the HT7 and TF4 resulted in significant reverse remodeling of the LA structure; thus, acupuncture is a viable option for reverse remodeling. There remains a lack of conclusive data on the reversal of LA structural remodeling after drug therapy in human studies, and there are no convenient or effective methods to reverse the process of pathological remodeling in the atria of patients with AF [22]. However, another hypothesis may exist for the mechanism of acupuncture in the treatment of AF. The modulatory effect of acupuncture on the autonomic system might have resulted in a reduction of triggers initiating AF, rather than promoting reverse remodeling. This possibility must be considered owing to the relatively short follow-up period in our study, and future studies on the mechanism of acupuncture on AF are warranted.

Unlike previous studies, the acupuncture treatment in our study was unable to reduce the recurrence rate of AF. There are several possible reasons for this finding, including that the small sample size may have resulted in insufficient power and the acupuncture protocol may not have been truly effective.

We made improvements to our own study design based on the limitations of previous trials. In previous studies, the participants were mostly patients with paroxysmal AF. One small randomized study showed that acupuncture at the points PC6, HT7, and BL15 may reduce AF recurrence in patients with persistent AF after cardioversion [10]. However, the study did not evaluate the combined effects of acupuncture and amiodarone, which is the standard treatment for AF. Another study provided seven consecutive days of acupuncture treatment at the point PC6 combined with amiodarone therapy for patients with persistent AF after catheter ablation. Additional treatment with acupuncture yielded AF recurrence rates lower than those of patients treated with amiodarone alone [8]. However, an add-on study design, which did not blind the participants or practitioners, may have influenced the results, and the study did not assess cardiac function using echocardiography.

### 4.3. Strengths and Limitations

This study has several strengths owing to improvements in the study design. To the best of our knowledge, this study is the first randomized, patient- and assessor-blinded prospective study to examine the efficacy and safety of acupuncture after EC on cardiac remodeling in patients with persistent AF. The participants in earlier studies were patients with paroxysmal AF, the studies were not double-blinded, and some studies only reported the therapeutic response, which is not an appropriate objective outcome variable in AF. In our trial, the patients were administered acupuncture treatment once a week, which is a more plausible treatment frequency, considering patients’ personal schedules (i.e., they cannot visit the clinic every day). To conduct a rigorous RCT, we used a strong sham control that required penetration of the skin. Other studies have used sham acupuncture, which only requires superficial placement of the needle. From a neurological perspective, penetration may induce more physiological reactions. Unfortunately, there is no consensus on which sham acupuncture is best; thus, our decision was based on other studies [6]. The type of sham control we selected allowed the control group to receive the same amount of attention and to minimize placebo effects. A qualitative study nested within our randomized trial revealed that blinding was successfully maintained [23].

However, our study also has a few limitations. One limitation was the relatively small number of patients, and hence the study findings from this study cannot be generalized to all patients with persistent AF. The sample size was also too small to draw definitive conclusions. The benefits of acupuncture may not be sound because of the small sample size and short follow-up duration. However, LA reverse remodeling is associated with favorable clinical outcomes, with no severe adverse events. Therefore, future studies with larger populations are required. In acupuncture related cardiology studies, conducting RCTs in which acupuncture treatment is performed on a strict schedule is difficult, and other studies have also reported similar limitations [10]. Qualitative interviews with patients revealed that doctor referrals were critical in improving patient recruitment. Consequently, we reached out to more clinicians instead of using bulletin boards, but the population that fit our inclusion criteria of having drug refractory, persistent AF was limited. Moreover, the use of several new acupuncture points for treatment and the use of a strong control group may have been a “double-edged sword”. It is not possible to explore the effects of every acupuncture point before a trial, but new points may have produced confounding effects that the researchers were unaware of. There were no significant differences between the two groups in the primary outcome. The present results need to be interpreted with caution due to the small sample size and short follow-up period. In future studies, we recommend following-up subjects for a longer period and increasing the number and duration of treatment. We also believe that drug refractory persistent atrial fibrillation patients may have been too advanced in clinical stages to respond significantly to acupuncture treatment and expanding the inclusion criteria may also be an option.

### 4.4. Implications for Future Study

In the future, due to the limitations mentioned above, larger randomized studies should be conducted to explore the mechanism and long-term effects of acupuncture on atrial remodeling. This study focused primarily on improving the methodological shortcomings of previous studies; for better results, utilizing a more pragmatic clinical design may be necessary. Studies with a comparative effectiveness research design for comparison of acupuncture treatments with already approved therapies might provide important information for practitioners and patients [24]. One previous study applied acupuncture for seven days and was able to reduce AF recurrence rate. Thus, consecutive treatment after cardioversion may be of critical importance to the therapeutic effect of acupuncture in patients with AF. As the dropout rate was relatively high in our study, we recommend consecutive acupuncture sessions with increased treatment frequency (more than once a week) and shortened treatment duration in future studies. Stavrakis et al. [9] used transcutaneous vagal stimulation. In the future studies, transcutaneous stimulation of acupuncture points by patients as a home treatment between clinical visits might be a possible option to increase the dosage of acupuncture treatment. In our nested qualitative study [23], patients who reported having AF symptoms had a more positive response to acupuncture treatment than those who did not. Narrowing inclusion criteria to include patients with AF symptoms may also improve the results.

Acupuncture is extremely safe, and there were no significant adverse events associated with treatment in this trial. The safety of acupuncture treatment in patients receiving a new oral anticoagulant has also been reported [25]. Therefore, providing standard acupuncture therapy is still a good option. Even though more rigorous evidence is required, consecutive acupuncture treatment after cardioversion should be considered.

As mentioned above, the small sample size, single-center study, and short follow-up period significantly limited the study. As a result, assessing the effect of acupuncture in AF patients requires caution. To reach definitive conclusions, large multicenter RCTs are required. To overcome these limitations, our research team also planned a retrospective nationwide cohort using National Health Insurance Service (NHIS) claim data [26]. With the RCTs and cohort study using NHIS data, we will be able to compensate for the shortcomings of the short follow-up period and the small sample size.

## 5. Conclusions

Patients in the acupuncture treatment group had more favorable atrial structural remodeling than those in the sham acupuncture treatment group, with no incidence of severe adverse events. However, acupuncture treatment after cardioversion did not change the rhythm-related outcomes after three months. Owing to the small sample size, these results are not conclusive. Further studies that adopt a comparative effectiveness design with a more intensive treatment frequency for patients with symptomatic AF are necessary to identify the mechanism and protective effect of acupuncture on cardiac electrophysiology in patients with AF.

## Figures and Tables

**Table 1:** Baseline characteristics and echocardiographic parameters.

	Sham Acupuncture (*N* = 15)	Acupuncture (*N* = 16)	*p*-Value
Age (years)	63.7 ± 6.5	69.6 ± 7.3	0.024
Male, *n* (%)	12 (80.0%)	13 (81.2%)	1
Hypertension, *n* (%)	10 (66.7%)	14 (87.5%)	0.339
Diabetes Mellitus, *n* (%)	3 (20.0%)	2 (12.5%)	0.937
Stroke, *n* (%)	0 (0.0%)	3 (18.8%)	0.247
Vascular disease, *n* (%)	0 (0.0%)	1 (6.2%)	1
CHA_2_DS_2_VASC score	1.6 ± 1.0	2.8 ± 1.5	0.02
BSA (kg/m^2^)	1.8 ± 0.2	1.8 ± 0.2	0.961
*TTE parameters*			
LVEF (%)	64.2 ± 5.3	61.0 ± 6.8	0.151
LAVI (mL/m^2^)	38.1 ± 7.7	42.2 ± 13.9	0.31
E/E’ *	11.5 [9.6–11.9]	12.6 [10.4–14.7]	0.455
Medications			
Beta blocker	1 (6.7%)	1 (6.2%)	1.0
Calcium channel blocker	3 (20%)	5 (31.2%)	0.685
ACEi	0	2 (12.5%)	0.484
ARB	5 (33.3%)	8 (50%)	0.347
ACEi or ARB	5 (33.3%)	10 (66.6%)	0.104
Loop diuretics	2 (13.3%)	1 (6.2%)	0.6
Thiazide	1 (6.7%)	0	0.484
Spironolactone	2 (13.3%)	2 (12.5%)	1.0
Any diuretics	3 (20%)	2 (12.5%)	0.654
Nitrate	1 (6.7%)	1 (6.2%)	1.0
Digitalis	1 (6.7%)	0	0.484
Anticoagulant			0.461
Warfarin	11 (73.3%)	13 (81.2%)	
DOACs	4 (26.7%)	3 (18.8%)	
(Apixaban)	0	1 (6.2%)	
(Dabigatran)	3 (20%)	1 (6.2%)	
(Edoxaban)	1 (6.7%)	0	
(Rivaroxaban)	0	1 (6.2%)	

Normally distributed data are expressed as mean ± standard deviation. * Non-normally distributed data are expressed as median (interquartile range). ACEi: Angiotensin-converting enzyme inhibitor, ARB: Angiotensin receptor blocker, AF: Atrial fibrillation, BSA: Body surface area, DOAC: Direct oral anticoagulants, E/E’: Mitral inflow velocity over septal mitral annulus tissue velocity, LAVI: Left atrial volume index, LVEF: Left ventricular ejection fraction, TTE: Transthoracic echocardiography, VKA: Vitamin K antagonists. Hypertension was defined as systolic blood pressure ≥ 140 mmHg or diastolic blood pressure ≥ 90 mmHg at more than two visits or a medical history of hypertension. Diabetes mellitus was defined as two or more of the following criteria: (1) having symptoms of diabetes (increased thirst, increased urination, and unexplained weight loss) and a blood glucose level ≥ 200 mg/dL; (2) fasting glucose level ≥ 126 mg/dL; (3) 2-h postprandial glucose ≥ 200 mg/dL by oral glucose tolerance test, hemoglobin A1_C_ ≥ 6.5%, or a medical history of diabetes mellitus. Stroke was confirmed by previous brain computed tomography and/or magnetic resonance imaging. No newly developed stroke occurred during the study period. Vascular disease was defined as previous myocardial infarction, peripheral artery disease, or aortic plaque.

**Table 2:** Clinical and echocardiographic outcomes three months after electrical cardioversion.

	Sham Acupuncture (*N* = 15)	Acupuncture (*N* = 16)	*p*-Value
Rhythm outcomes			0.611
AF recurrence	9 (60.0%)	12 (75.0%)	
Sinus rhythm	6 (40.0%)	4 (25.0%)	
TTE parameters			
LVEF	63.8 ± 4.6	63.6 ± 5.2	0.903
LAVI	36.9 ± 14.1	36.1 ± 9.7	0.865
E/E’	11.9 ± 4.5	11.9 ± 3.9	0.984

AF: Atrial fibrillation, E/E’: Mitral inflow velocity over septal mitral annulus tissue velocity, LAVI: Left atrial volume index, LVEF: Left ventricular ejection fraction, TTE: Transthoracic echocardiography.

## Data Availability

Data are available on request to corresponding author.

## Supplementary Materials

The following are available online at https://www.mdpi.com/article/10.3390/medicina58010041/s1. Table S1: Details of acupuncture treatments based on the STRICTA 2010 checklist, Table S2: Details of acupuncture points * in the active and sham acupuncture treatments, Table S3: CONSORT 2010 checklist of information to include when reporting a randomised trial.

## Abbreviations

ACEi: Angiotensin-converting enzyme inhibitor, ARB: Angiotensin receptor blocker, AF: Atrial fibrillation, EA: Electroacupuncture, BSA: Body surface area, E/E’: Mitral inflow velocity over septal mitral annulus tissue velocity, EC: Electrical cardioversion, ECG: Electrocardiography, IDA: Intradermal acupuncture, LA: Left atrium, LAA: Left atrial appendage, LAVI: Left atrial volume index, LV: Left ventricular, LVEF: Left ventricular ejection fraction, MI: Myocardial infarction, NHIS: National health insurance service, TEE: Transesophageal echocardiography, TTE: Transthoracic echocardiography.
