# Supplementary material for: Effects of Acupuncture on Cardiac Remodeling in Patients with Persistent Atrial Fibrillation: Results of a Randomized, Placebo-Controlled, Patient- and Assessor-Blinded Pilot Trial and Its Implications for Future Research
Source: Medicina (Kaunas). 2021 Dec 27;58(1):41. doi: 10.3390/medicina58010041 (PMC8778603; doi:10.3390/medicina58010041)
Supplement: Supplementary file 1 [file medicina-58-00041-s001.zip › Supplemental Table S1.pdf]

Supplement Table S1. Details of acupuncture treatments based on the STRICTA 2010 checklist

Item		Detail
**1. Acupuncture rationale**	**1a) Style of acupuncture**	Electroacupuncture and intradermal acupuncture based on traditional Korean Medicine theory
	**1b) Reasoning for treatment provided, based on historical context, literature sources, and/or consensus methods, with references where appropriate**	Based on consensus of the Korean medicine doctors, clinical experience, and experimental/clinical articles
	**1c) Extent to which treatment was varied**	All participants received standardized treatment
**2.Details of needling**	**2a) Number of needle insertions per subject per session**	4 electroacupuncture needles & 4 intradermal acupuncture needles
	**2b) Names of points used**	Lt PC5, Lt PC6, Lt ST36, Lt ST37 (4 points for electroacupuncture)<br>Both HT7 , both TF4 (4 points for intradermal acupuncture)
	**2c) Depth of insertion, based on a specified unit of measurement**	2.0 ± 0.5 cm (electroacupuncture)<br>About 1 mm (intradermal acupuncture)
	**2d) Response sought**	De-qi and slight muscle twitch
	**2e) Needle stimulation**<br>:	1) 3 times clockwise rotated to induce de-qi sensation<br>2) Electrical stimulation with a frequency of 2 Hz, 400 μs per stimulation (ES-160, ITO, Japan)
	**2f) Needle retention time**	20 minutes
	**2g) Needle type**	Acupuncture : disposable, sterile needles (0.20mm × 30mm, Dongbang Acupuncture Inc., Boryung, Korea)<br>Intradermal acupuncture : disposable, sterile, sticker-type

		intredermal needle (0.18 × 1.3 × 1.5 mm, Dongbang Acupuncture Inc., Boryung, Korea)
**3. Treatment regimen**	**3a) Number of treatment sessions**	10 session
	**3b) Frequency and duration of treatment sessions**	10 acupuncture treatments session over 10 weeks. It took 20 minutes per each session
**4. Other components of treatment**	**4a) Details of other interventions administered to the acupuncture group**	Conventional western medicine management according to atrial fibrillation management guideline
	**4b) Setting and context of treatment, including instructions to practitioners, and information and explanations to patients**	All patients were admitted to the cardiac ward for treatment of atrial fibrillation by electronic cardioversion. All information was provided to participants. Follow up acupuncture treatment was conducted in outpatient department.
**5. Practitioner background**	**5) Description of participating acupuncturists**	Licensed Korean medicine doctor with at least 3 years of clinical practice. The practitioners have studied acupuncture for more than 6 years and graduated from the College of Korean Medicine. The Korean medicine doctor was licensed by Korean Minisity of Health and Welfare. Standardized operation procedures were predefined for reliable precedure
**6. Control interventions**	**6a) Rationale for the control or comparator in the context of the research question, with sources that justify this choice**	Based on consensus of the Korean medicine doctors, clinical experience, and experimental/clinical articles
	**6b) Precise description of the control or comparator. If sham acupuncture or any other type of acupuncture-like control is used, provide details as for Items 1 to 3 above.**	Acupuncture point : See Supplement Table 2<br><br>Depth of insertion : 0.5cm<br><br>De-qi sensation and electronic current was not adopted.<br><br>Other aspect of sham treatment was similar to active acupuncture group.
