# Supplementary material for: Effects of Acupuncture on Cardiac Remodeling in Patients with Persistent Atrial Fibrillation: Results of a Randomized, Placebo-Controlled, Patient- and Assessor-Blinded Pilot Trial and Its Implications for Future Research
Source: Medicina (Kaunas). 2021 Dec 27;58(1):41. doi: 10.3390/medicina58010041 (PMC8778603; doi:10.3390/medicina58010041)
Supplement: Supplementary file 1 [file medicina-58-00041-s001.zip › Supplemental Table S2.pdf]

Supplement Table S2. Details of Acupuncture Points* in Active and Sham Acupuncture Treatment

	Active acupuncture treatment	Sham acupuncture treatment
EA	PC5	lateral 1.5 cm to PC5 (between PC and LU meridians)
	PC6	lateral 1.5 cm to PC6 (between PC and LU meridians)
	ST36	lateral 2 cm to ST36 (between ST and GB meridians)
	ST37	lateral 2 cm to ST37 (between ST and GB meridians)
IDA	HT7	lateral to the 2nd extensor digitorum tendon (between TE4 and LI5)
	TF4	between the jaw point and clavicle point in ear acupuncture

* Acupuncture point PC5 refers to the 5th point of pericardium meridian, and ear acupuncture points have different nomenclature (e.g., TF4 means the 4th point in triangular fossa).

EA = electroacupuncture; GB = gall bladder; IDA = intradermal acupuncture; LI = large intestine; LU = lung; PC = pericardium; ST = stomach; TE = triple energizer.
